# The Safety and Efficacy of Rituximab and Belimumab in Systemic Lupus Erythematosus: A Systematic Review

**DOI:** 10.7759/cureus.40719

**Published:** 2023-06-21

**Authors:** Naushad Abid, Sara Manaye, Hamzah Naushad, Kaaviya Cheran, Chinmayee Murthy, Elisa A Bornemann, Hari Krishna Kamma, Mohammad Alabbas, Mohammed Elashahab

**Affiliations:** 1 Internal Medicine, King Faisal University College of Medicine, Al-Ahsa, SAU; 2 Internal Medicine / Rheumatology, King Faisal University, Al-Ahsa, SAU; 3 Internal Medicine, California Institute of Behavioral Neurosciences & Psychology, Fairfield, USA; 4 Medicine and Surgery, Dow International Medical College, Dow University of Health Sciences, Karachi, PAK; 5 Internal Medicine, Bidar Institute of Medical Sciences, Bidar, IND; 6 Medicine and Surgery, Universidad Latina de Panama, Panama City, PAN; 7 Psychiatry, California Institute of Behavioral Neurosciences & Psychology, Fairfield, USA; 8 Internal Medicine, Faculty of Medicine, University of Debrecen, Debrecen, HUN

**Keywords:** b-cell biology, lupus nephritis flare, systemic lupus, rituximab, belimumab

## Abstract

There is a vital role of B cells in the pathogenesis of Systemic Lupus Erythematosus (SLE). Belimumab (Bel), an inhibitor of B cell activating factor (BAFF), and Rituximab (RTX), a monoclonal antibody targeting Cd20 antigen, have been used to manage systemic lupus. Several randomized controlled trials (RCTs) have evaluated these two agents' clinical efficacy and safety in different manifestations of SLE. This study aims to review the randomized control trials involving these two agents systematically and to explain if any disparity is noticed in the primary and secondary outcomes between these two agents. This study is done according to Preferred Reporting Items for Systematic Reviews and Meta-Analyses (PRISMA) guidelines. After applying the inclusion criteria and quality assessment by independent reviewers and co-authors, relevant papers were identified, and data were extracted. The results have shown that RCTs involving Belimumab achieved primary endpoints; however, targeted endpoints were not achieved in studies involving Rituximab. It is concluded that despite the conflicting results obtained in clinical trials, both are effective in systemic lupus, as indicated in real-world clinical experience. However, better-designed multicenter studies evaluating these B-cell-targeting drugs are needed.

## Introduction and background

Systemic lupus erythematosus (SLE) is an autoimmune disease with a chronic relapsing-remitting course and variable manifestations ranging from mild illness to life-threatening multiorgan involvement. Pathogenesis involves genetic predisposition interacting with environmental, immunological, and hormonal factors [1-3].

Patients with SLE continue to experience considerable morbidity and mortality despite significant advancements in treatment [4-6]. A typical active patient with SLE is managed with multiple immunosuppressive medications, which control the disease activity and increase the risk of severe side effects [7,8]. SLE is a challenging disease to treat due to its complex pathophysiology that may be difficult to control with a single agent. The management is further complicated as the majority of the patient are young females of childbearing age, and there are increasing consequences regarding teratogenicity, infertility, and carcinogenicity with immunosuppressive medications particularly with Cyclophosphamide (CYC), when used in higher than usual doses. Renal involvement, a significant cause of morbidity and mortality, eventually develops in 50% of these patients and is a marker of poor prognosis [9].

There has been overwhelming evidence that B cells play a crucial role in disease pathogenesis, as shown in the mice model [10, 11]. B cells pass through various stages before forming antibody-producing plasma cells and during this process express various cell surface antigen such as CD20 and CD22. Additionally, they also produce pro-inflammatory cytokines and regulate T-cell activity [12]. The loss of self-tolerance in B cell development and activity can lead to autoantibodies production [12]. As B cell dysfunction has been linked directly and indirectly to autoimmunity, there has been considerable interest in B cell-targeted therapy in managing SLE [13]. An anti-CD20 monoclonal antibody (mAb), Rituximab, has been tried in SLE and in other Rheumatologic conditions such as vasculitis [13]. However, SLE treatment failures have been observed even after targeting B-cell-surface antigen, which led to alternative targets of B-cell activation, such as B lymphocyte stimulator (BlyS) or B cell-activating factor (BAFF) [14]. Belimumab, a monoclonal antibody against BlyS has successfully completed phase III trials [14, 15].

Various randomized control trials have been published in the literature evaluating the efficacy of these two B cells targeting agents in renal and non-renal lupus. Our study aims to analyze and compare the efficacy and safety profile of these two drugs in systemic lupus and to explain if any possible disparities are noticed in the primary and secondary outcomes between these two agents.

## Review

Methodology

**Figure 1:**
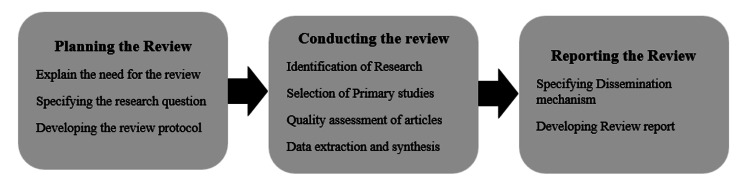
Summary of the steps involved in our review

In this study, we have followed the systematic literature review guidelines recommended by Preferred Reporting Items for Systematic Reviews and Meta-Analyses (PRISMA) [16], and methods proposed by Muka et al. [17]. Figure 1 summarizes each step which includes planning, conducting and reporting the systemic review. Additionally, we give details of actions as they were performed for this systematic review.

Planning the Review

Planning of the review includes justification of the need of the review, generating research questions using PICO (Population, intervention, comparison and outcome framework) and formulating a search strategy.

Formulating a search strategy: We identified eligible studies by searching MEDLINE via PubMed, PubMed Central, and Google Scholar from its inception to September 2022. Keywords were identified using three concepts in our review (Belimumab, Rituximab, Systemic lupus erythematosus). These Keywords were used to generate the medical subject headings (Mesh) in PubMed. Finally, the medical subject heading (Mesh) terms along with Boolean logical operators were combined to come up with search strategy. Table 1 summarizes the steps.

**Table 1:** Summary of search strategy used in our review

Keywords	Medical subject headings (Mesh)	Query strings	Final Search Term-Combining query strings with Boolean logical operators
Belimumab OR B cell depleting agents	"belimumab" [Supplementary Concept]	Belimumab OR B cell depleting agents OR"belimumab" [Supplementary Concept]	Belimumab OR B cell depleting agents OR"belimumab" [Supplementary Concept] OR RITUXIMAB OR cd20 antibody OR (“Rituximab/adverse effects"[Mesh] OR “Rituximab/drug effects"[Mesh] OR “Rituximab/therapeutic use"[Mesh] ) AND Systemic lupus Erythematosus OR "Lupus Erythematosus, Systemic/drug therapy"[Majr]
RITUXIMAB OR Cd20 antibody	(“Rituximab/adverse effects"[Mesh] OR “Rituximab/drug effects"[Mesh] OR "Rituximab/therapeutic use"[Mesh]	RITUXIMAB OR anti cd20 antibody OR (“Rituximab/adverse effects"[Mesh] OR “Rituximab/drug effects"[Mesh] OR “Rituximab/therapeutic use"[Mesh]	
Systemic Lupus Erythematosus	("Lupus Erythematosus, Systemic/drug therapy"[Mesh] OR "Lupus Erythematosus, Systemic/therapy"[Mesh]	Systemic Lupus Erythematosus OR "Lupus Erythematosus, Systemic/drug therapy"[Majr]	

Inclusion criteria: Eligibility criteria in this systematic review included randomized controlled trials, Open-Label Extension studies of randomized controlled trials conducted on adults (18 years and above) and published in the English language in peer-reviewed journals.

Exclusion criteria: Non-randomized clinical trials, case reports, concise reports, reviews and gray literature were not included.

Quality assessment: The Cochrane Collaboration tool [18] was used to assess the methodological quality and the risk of bias of the selected studies. This tool used the domain-based evaluation ("+," low risk of bias; "−," high risk of bias; "?", unclear risk of bias). The summary of the quality assessment using this tool is summarised in Table 2 below.

**Table 2:** Cochrane collaboration's tool for assessing the risk of bias

Bias Domain	Source of Bias	EXPLORER Trial	LUNAR Trial	BLISS 52 Trial	BLISS 76 Trial	BLISS SC Trial	EMBRACE Trial	BLISS LN Trial	CALIBRATE Trial	YOSHIKA et al.	ZHANG et al.
Selection Bias	Random Sequence Generation	+	+	+	+	+	+	+	+	+	+
	Allocation Concealment	+	+	+	+	+	+	+	+	+	+
Performance Bias	Blinding of participants and personnel	+	+	+	+	+	+	+	OPEN LABEL	+	+
Detection Bias	Blinding of outcome assessment	+	+	+	+	+	+	+	+	+	+
Attrition Bias	Incomplete outcome data	+	+	+	+	+	+	+	+	+	+
Reporting Bias	Selective reporting	+	+	+	+	+	+	+	+	+	+
Other Bias	Anything else, ideally prespecified	+	+	+	+	+	+	+	+	+	+

Conducting the Review

**Figure 2:**
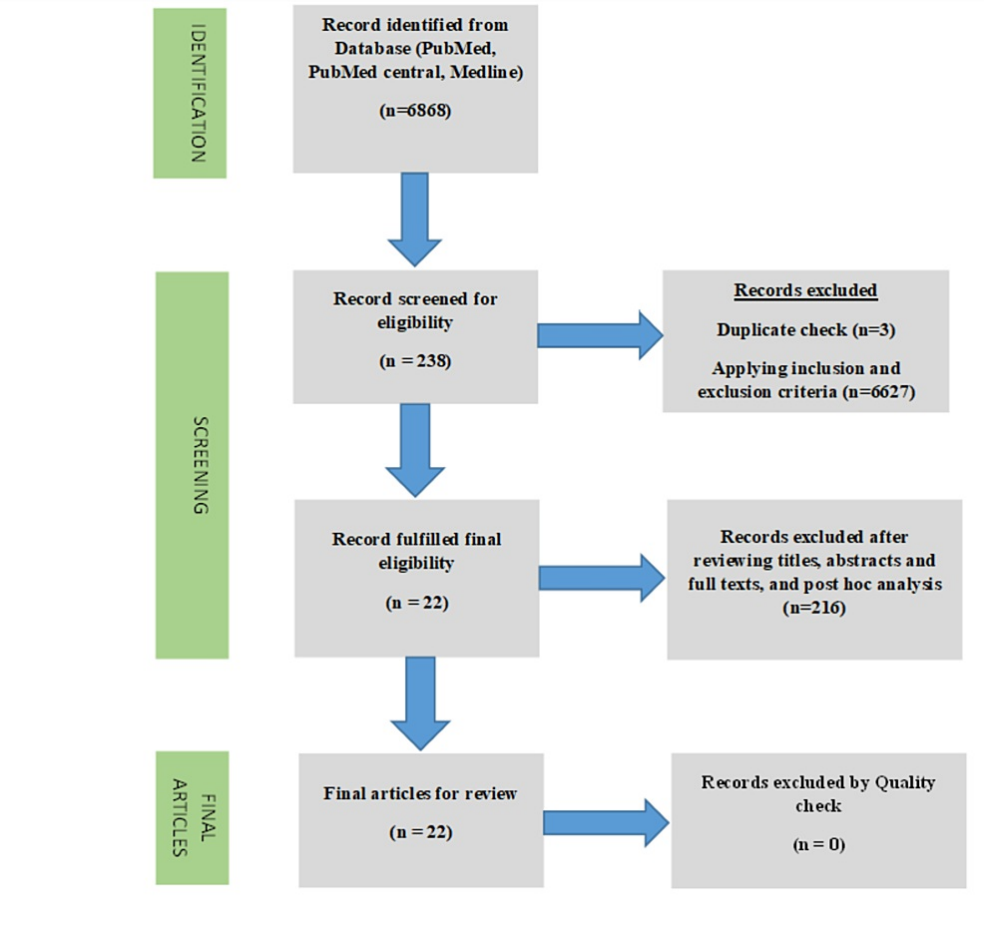
PRISMA Flow diagram with description of the process in paper selection

We adopted a systematic process using PRISMA guidelines for study selection, as shown using a PRISMA flow diagram in Figure 2.

Results

The initial search resulted in 6868 papers. After duplicate identification and applying inclusion and exclusion criteria, 238 papers were selected for screening. Two independent reviewers and authors screened 238 papers. Study selection was based on the title and abstract, and full-text articles. After screening by reviewing titles, abstracts, and full-text articles, 22 papers were selected and subjected to quality check (Table 1) by a team comprising of authors and two external reviewers. All 22 articles were eligible for review after discussion and evaluation by team members.

Reporting the Review

The last step in a systematic review is writing and presenting it to the medical community. Therefore, after a thorough literature review, we tried to answer all the Research questions in the introduction and discussion.

Discussion

The future of SLE patients is promising due to a better understanding of the disease's pathogenesis and advancements in immunosuppressive medications. Currently, B-cell-targeted therapies are the new strategies for managing SLE patients. Rituximab (RTX), an anti-CD20 mAb, and Belimumab (Bel), a mAb directed against B cell activating factor (BAFF), have been explored in randomized controlled trials (RCTs) [14, 15].

In this section, we will discuss the safety and efficacy of Rituximab and Belimumab from the randomized controlled trials (RCTs), the open-label extension of some of these (RCTs), followed by a critical review of the paradox of Rituximab and Belimumab.

Rituximab

Clinical efficacy: The clinical efficacy of RTX was evaluated using the Exploratory Phase II/III SLE Evaluation of Rituximab, (EXPLORER) trial [19] and the Lupus Nephritis Assessment with Rituximab (LUNAR) trial [20].

The EXPLORER trial [19] tested the efficacy and safety of Rituximab. Patients (n=257) were identified as having moderate to severe SLE by ≥1 British Isles Lupus Assessment Group (BILAG) A score or ≥2 BILAG B scores. All selected patients continued baseline immunosuppressive medication and were randomized at a ratio of 2:1 to receive Rituximab (1,000 mg) or placebo. The trial's primary endpoint was placebo versus Rituximab's effect in achieving and maintaining a significant clinical response, a partial clinical response, or no clinical response at week 52 using each BILAG index organ system scores. No differences between placebo and Rituximab were observed in the primary and secondary efficacy endpoints, including the BILAG-defined response. The treatment group, however, demonstrated beneficial effects in the African American and Hispanic subgroups.

Lupus Nephritis Assessment with Rituximab LUNAR was a double-blind, placebo-controlled trial [20] that tested the efficacy of Rituximab in patients with class *111 *and *1V* lupus nephritis. The patients were randomized in a 1:1 pattern to receive Rituximab or placebo concomitantly with mycophenolate mofetil (MMF) and corticosteroids. The primary end was renal response status at week 52. Results revealed that the renal response rates among the treatment and placebo groups were insignificant (45.8 vs. 56.9%, p-value = .018). However, despite failing to get the primary endpoint, the Rituximab treated group showed significant improvement in complement and anti-dsDNA levels.

In summary, primary endpoints were not achieved with both trials; however, Rituximab was shown to reduce the frequency of flares in the EXPLORER trial, and improved serologic markers were seen in the LUNAR trial.

Table 3 summarizes the RCTs related to Rituximab and their significant findings.

**Table 3:** Summary of the trials involving Rituximab

Drug	Significant Trials	Findings
Rituximab – Chimeric anti-CD20 monoclonal antibody	EXPLORER TRIAL [19]	-Patients with moderate to severe SLE did not achieve primary endpoint. -Showed reduced risk and frequency of flares - A beneficial effect of the primary endpoint was observed in the African American and Hispanic subgroups.
	LUNAR TRIAL [20]	-Patients with class *111 *or *1V* lupus nephritis were randomized. -Primary endpoint was not achieved. - Significant improvement in complement levels and Anti-DsDNA levels were observed among the Rituximab treated group.

Safety of Rituximab

EXPLORER TRIAL: Adverse events were similar in the two groups (37.9% vs. 36.4%) [19]. Most were upper respiratory tract infections and infusion-related adverse events. One patient in the placebo group died due to cardiopulmonary arrest. In contrast, three patients in the Rituximab group died, including one patient with a perforated bowel, one patient with multiple drug intoxications, and one in whom the cause of death was unknown.

LUNAR TRIAL [20]: Respiratory tract infections and infusion-related reactions were seen in the treatment and placebo groups. Two deaths occurred in the Rituximab group, and both were considered unrelated to Rituximab. One elderly patient had Staphylococcus aureus-related sepsis more than 60 days after the Rituximab infusion. In contrast, another death occurred in a young female due to alveolar hemorrhage, which developed 58 days after the drug infusion.

Patients on Rituximab can develop human anti-chimeric antibodies (HACA). Serum sickness and severe infusion-related effects due to HACA occurred in three patients in EXPLORER and one in LUNAR TRIAL, respectively.

No patient in these trials developed progressive multifocal leukoencephalopathy, though some cases are reported in the literature.

Belimumab (Bel):

Clinical efficacy: Belimumab (Bel) is a fully-humanized monoclonal antibody against BlyS. Belimumab is FDA-approved for the treatment of moderately active SLE.

BLISS 52 (n=865) and BLISS 76 (n=819) [21,22] are two multicenter RCTs involving adult patients with active SLE (excluding patients with lupus nephritis and CNS involvement). All included patients were randomly assigned to receive IV Belimumab (IV Bel) 1, 10 mg/kg, or placebo and were continued on Standard of Care (SOC). Additionally, all the patients were required to be on corticosteroids, non-steroidal anti-inflammatory, anti-malarial, and other immunosuppressive medications. The primary endpoints included various scoring systems to assess the disease activity, such as Physician Global Assessment (PGA), Systemic Lupus Erythematous Responder Index (SRI)-4 score (defined as >4-point reduction in the Safety of Estrogens in Lupus Erythematous National Assessment-Systemic Lupus Erythematosus Disease Activity Index (SELENA-SLEDAI), British Isles Lupus Activity Group (BILAG) A organ domain score, and BILAG B score. The secondary endpoints included disease activity scores (SRI-4 and PGA) along with the percentage of patients with a mean prednisone dose reduction of >25% from baseline and <7.5 mg/day. The trial achieved a statistically significant SRI 4 response over placebo in both arms of Belimumab. Additionally, in comparison to the placebo, a reduction in SLE disease activity and flare recurrence was noticed.

Another trial evaluated subcutaneous (SC) Belimumab in SLE (BLISS -SC Trial) [23]. SLE patients with moderate to severe activity (a SELENA-SLEDAI score of >8) were continued on SLE treatment and randomized for 52 weeks in a 2:1 ratio to receive weekly SC Belimumab 200 mg or placebo. The primary endpoint was the disease activity assessment using SRI 4 at the 52nd week, and the secondary endpoints were a reduction in the corticosteroid dosage and the onset of a new flare-up. The SRI-4 response at week 52 was met by a statistically significant number of patients in the Belimumab arm compared to the placebo arm (61.4% vs. 48.4%, respectively). The addition of subcutaneous Belimumab to a Standard of Care (SOC) SLE medication significantly decreased severe disease flare-ups and allowed for steroid tapering.

Zhang et al. [24] conducted a phase III randomized, double-blind, placebo-controlled study in centers across China and Japan. Patients with SLE were randomized for 48 weeks into intravenous Belimumab 10 mg/kg or placebo, plus SOC treatment every four weeks. The primary and secondary endpoints were almost like BLISS 52 and 76 trials. At week 52, the SRI4 response rate was higher with Belimumab versus Placebo (53.8% vs. 40.1%; P=0.0001). The percentage of patients with SRI 4 and SRI7 response was significantly more significant for Belimumab versus placebo. Compared to placebo, patients in the Belimumab group had a 50% lower risk of experiencing a severe flare. In a similar pattern of trials in Japanese patients [25] with SLE, Belimumab showed improved disease activity.

As patients of black and Hispanic ethnicity were not represented well in previous trials, so EMBRACE trial was conducted [26]. This study assessed the efficacy and safety of intravenous (IV) Belimumab plus standard therapy in patients of Black African Ancestry. EMBRACE was a 52-week double-blind, placebo-controlled trial conducted in Black adults with active SLE. The patients were continued on standard treatment and were randomized to receive monthly Belimumab 10 mg/kg IV or placebo. The primary endpoint of the study was the SRI response rate at week 52 and a proteinuria scoring derived from the SLE Disease Activity Index 2000 (SLEDAI-2K). The secondary endpoints included the SRI response rate at week 52 and reductions in prednisone dose. The primary endpoints were not achieved. However, patients on Bel were able to taper corticosteroids, making it a possibly suitable option for SLE management in this population.

BLISS 52 and 76 did not assess the safety and efficacy of BEL in lupus nephritis, so Belimumab International Study in Lupus Nephritis (BLISS-LN) was conducted [27]. This was a phase III, multinational, randomized, double-blind, placebo-controlled trial. Patients with active lupus nephritis (n=448), in addition to standard therapy including mycophenolate mofetil (MMF) or cyclophosphamide-azathioprine, were assigned to receive intravenous Belimumab (at a dose of 10 mg per kilogram of body weight) or placebo. The primary endpoint measured renal efficacy parameters such as urine protein/creatinine ratio, and a pre-defined estimated glomerular filtration rate. After a study period of 104 weeks, significantly more patients in the Belimumab group than in the placebo group had a primary efficacy renal response (43% vs. 32%; odds ratio, 1.6;) and a complete renal response (30% vs. 20%; odds ratio, 1.7; 95% CI, 1.1 to 2.7). The reaction in the Belimumab cohort was better than the placebo if the induction therapy was high-dose corticosteroids (HDCS) and MMF but not HDCS and CYC. The patients of African American ancestry who received Belimumab appeared to be more likely to achieve primary efficacy renal response and complete renal response.

Table 4 gives the summary of RCTs involving Belimumab.

**Table 4:** Summary of major trials involving Belimumab

Drug	Significant Trials	Findings
Belimumab: Human anti-BlyS monoclonal antibody (intravenous or subcutaneous)	BLISS-52 and BLISS-76 [21,22]	-Multicenter, double-blind placebo-controlled trials. -Primary endpoints were achieved in both with significant improvement in SRI scores in both. -Reduced Corticosteroid dosage and disease flare and activity were observed. -Subgroup analysis revealed improvement in Lupus nephritis
	BLISS-LN [27]	This was a phase 3, multinational, randomized, double-blind, placebo-controlled involving patients with active nephritis. -Primary endpoint was achieved after induction treatment with HDCS and MMF in lupus nephritis class *111, 1V*.
	EMBRACE Trial [26]	A multicenter, double-blind, placebo-controlled trial in adults of Black race with active SLE. -The primary endpoint was not achieved - Corticosteroid tapering was possible in BEL group

RCTs Evaluating the Combination of Rituximab and Belimumab

Rituximab spares pre-B cells and plasma cells as their target antigen; Cd20 is not expressed in these cells leading to the development of memory B cells and disease flares after Rituximab treatment. Additionally, levels of Blys are elevated following treatment with Rituximab, so administering Belimumab after Rituximab is likely to prevent the emergence of auto-reactive B cells.

CALIBRATE trial [28] was a randomized open-label clinical trial which assessed the efficacy of RTX followed by Bel in patients with lupus nephritis. Patients (n=43) were randomly divided into two groups. The first group patients were treated with Rituximab, cyclophosphamide (CYC), and glucocorticoids, followed by Bel infusions (RCB group) and the other group received only Rituximab and cyclophosphamide only (RC group). The percentages of B cell subsets in the blood were analyzed at week 96. The trial's primary endpoint was the safety of the trial protocol, defined as having at least one serious infection during the study period. The study showed that treatment with Bel did not increase the incidence of infections. At week 48, renal response occurred in 52% of 21 patients receiving Belimumab, compared to 41% of 22 patients in the RC group (p=NS). B cells depletion happened in both groups, though much lower in the Bel group. It was concluded that effects on B cells were demonstrated in patients receiving Bel, but no significant clinical benefit was achieved on adding Bel in study subjects.

Adverse Effects (AE) of Belimumab in RCTs

Adverse effects noticed with intravenous and subcutaneous forms of Belimumab were almost comparable.

In BLISS 76 trial [22], profound and severe AEs, including infections, malignancies, and deaths, were similar in all groups. Three deaths occurred in patients receiving Belimumab during the study; none were related to Belimumab. Depression was noticed frequently in the Belimumab group compared to placebo (6% to 4% respectively); however, suicide or suicide attempts were not seen in any group. Severe infections occurred at similar rates in all treatment groups. There was no mortality associated with severe illness. Only one patient had disseminated cytomegalovirus infection, which was treated. Malignancies of different origins were seen mainly in the Belimumab group, but the rate was not higher than the reported cases in the literature for SLE.

In BLISS 52 [21], trial rates of adverse events were similar in all the groups.

In the BLISS LN trial [27], side effects profile for Belimumab plus standard therapy was like that of traditional treatment alone. However, 11 patients died during the trial (six in the Belimumab group and five in the placebo group), and none of them was related to Belimumab.

In the BLISS SC TRIAL [23], 449 patients in the Belimumab group (80.8%) and 236 patients in the placebo group (84.3%) experienced at least one AE. The most common AEs were infections. Serious adverse effects (SAEs) were reported for 10.8% and 15.7% of patients. Local injection site reactions and hypersensitivity reactions were seen equally in both groups. Depression was noticed in the Belimumab group and placebo (2.6% vs. 3.7%, respectively). Two cases of severe suicidal ideation (0.4% taking Belimumab) were reported.

In the EMBRACE TRIAL [26], the AEs most reported in either treatment group during the double-blind phase were upper respiratory and urinary tract infections. Two patients in the Belimumab group (0.6%) died, and none was considered related to Belimumab.

In RCT from Japan [25], a higher incidence of AEs was observed in the Belimumab group (100.0%) compared with the placebo group (90.5%). The most common AE reported in both groups was nasopharyngitis. However, a higher percentage of patients experienced depression in the Belimumab group (7.7%) compared with the placebo group (4.8%).

Overview of adverse effects related to Belimumab

Infections and lack of response appeared to be the common reason for drug discontinuation. The adverse events included infections, infusion reactions, hypersensitivity, headache, nausea, and fatigue. Additionally, psychiatric disorders were seen in 16% of patients, especially those with preceding history of psychiatric disease [29]. The drug appeared to be safe in most trials; however, the history of depression and suicidal ideation should be explored before starting Belimumab [29].

Trials Evaluating Long-Term Safety

In the literature review, we found some studies which were the continuation of Phase III RCTs involving Belimumab only. No long-term safety studies involving Rituximab were found.

Furie et al. [30] assessed Belimumab's long-term safety and efficacy in individuals who completed the BLISS 76 trial. Patients continued to receive the exact Belimumab dosage along with standard treatment, and those in the placebo group were given Belimumab at a dose of 10 mg/kg. The primary outcome measure was disease activity assessment using the Systemic Lupus International Collaborating Clinics/American College of Rheumatology Damage Index (SDI). Results revealed that the overall incidence of severe and treatment-related AEs remained stable or declined. In addition, the improvement in SRI and reduction in SELENA / SLEDAI were observed.

In another open-label continuation study (n=296) of the BLISS 52 trial, the results revealed that safety profile and drug efficacy were maintained throughout seven years of the study [31].

Bruce et al. [29] have examined long-term organ damage and safety following Belimumab plus standard of Care (SOC) treatment. Data was examined from two ongoing open-label studies that enrolled patients who completed BLISS-52 or BLISS-76. Patients received Belimumab every four weeks plus SOC, and disease activity was assessed using SLICC Damage Index (SDI). It was concluded that patients with SLE treated with Belimumab, and SOC have minor organ damage and adverse effects [29].

Pooled data from BLISS 52 AND 76 trials also showed normalization of serologic activity and reduction in BLyS-dependent B-cell subsets in clinically active patients. Significant improvement in health-related quality of life (HQRL) parameters was also observed, which correlated with improvement in disease activity [32,33].

A subgroup analysis from Japan demonstrated organ system improvements in Japanese Belimumab-treated patients [34]. In addition, a recently open-label extension of phase III RCT from China revealed long-term safety [35].

In summary, open-label extension, and pooled data analysis of RCTs have demonstrated Belimumab's long-term safety and efficacy in clinically active SLE.

Rituximab and Belimumab Paradox

Although both Belimumab and Rituximab are B cells depleting drugs, the disparity is seen in the results of RCTs done on these drugs. Wise and Stohl [14], in their review, have investigated and named this as the "RTX/BEL paradox". According to them, trial design, SLE phenotype, and the factors related to B cells could contribute.

There was a difference in the structure of RCTs done for Bel and RTX. The EXPLORER trial allowed unrestricted use of corticosteroids, which may have led to a favorable clinical response in the placebo arm, masking a statistically significant difference between the treatment and placebo group. Similarly, the LUNAR trial allowed concurrent use of MMF, the primary drug used in lupus nephritis, which might have masked the significant difference between the two groups. In addition, the primary outcome measure used in the LUNAR trial might be too restrictive to detect any significance. In contrast, SRI4 was used in Bel-related RCTs, and it was sensitive to pick minor differences between treatment and placebo groups. A favorable outcome could have been achieved by enrolling a more significant number of patients in RCTs involving RTX [14,21-22].

SLE phenotype might have contributed to RTX/Bel paradox. For example, SLE patients in clinical settings subjected to RTX may have B cell-driven aggressive disease, which indicates their response to RTX, as seen in African American patients. In contrast, those with mild phenotype, as seen in RCTs (in patients of European descent), may have mild disease indicating less responsiveness to RTX [14].

There are some subsets of B cells which are called B regulatory cells (Breg), and they help to control the dysregulated immune response. Therefore, it is likely that RTX induces significant B cell depletion, thereby depleting the Breg cells and causing a diminished clinical response in SLE patients. In contrast, Bel preferentially downregulates autoreactive (pathogenic) B cells while sparing the Breg, explaining the beneficial response observed with them in RCTs [36].

Additionally, the CD20 antigen, the main target of RTX, is not expressed in plasma cells, which may allow continuous autoantibody production even after adding RTX. On the other hand, Bel can also target plasma cells contributing to its success in clinical trials [37]. Belimumab also has been shown to modulate non-B-cell elements of the immune system that contribute to SLE activity, whereas RTX is B-cell specific, which further explains the RTX/BEL paradox [38].

## Conclusions

There is no doubt that B cells targeting therapies are the cornerstone in managing systemic lupus. Although RCTs failed to achieve primary endpoints for RTX, depletion of CD20-positive cells has shown promising results in clinical settings. High levels of BAFF in active SLE patients make its inhibition by Bel a logical choice, as demonstrated by phase III clinical trials and long-term follow-up studies. However, it still needs to be clarified which group of patients with active SLE would benefit from B cell-depleting drugs. In the case of Belimumab, phase III clinical trials indicate that SLE patients with the active disease having mucocutaneous, musculoskeletal, and active lupus nephritis would benefit most. However, the duration of treatment with Belimumab still needs to be clarified. There is always an ongoing debate regarding using RTX, but clinical experiences have shown promising results with RTX in refractory nephritis and neuropsychiatric lupus.

Combining the two drugs might have synergistic effects, as shown in CALIBRATE trial. However, administration of Bel before RTX will also target memory B cells from the tissue and facilitate more significant effects with Rituximab. There are some ongoing trials assessing the efficacy and safety of adding Bel before RTX but results are not yet published in the literature.
